# ICU Clinicians' View on Platelet Transfusion Thresholds for a Future Trial—Protocol for an International Survey

**DOI:** 10.1111/aas.70263

**Published:** 2026-05-19

**Authors:** Jehad Ahmad Barakji, Carl Thomas Anthon, Anders Granholm, Morten Hylander Møller, Jakob Stensballe, Anders Perner

**Affiliations:** ^1^ Department of Intensive Care Copenhagen University Hospital—Rigshospitalet Copenhagen Denmark; ^2^ Section of Biostatistics, Department of Public Health University of Copenhagen Copenhagen Denmark; ^3^ Department of Clinical Medicine University of Copenhagen Copenhagen Denmark; ^4^ Department of Anaesthesiology, Surgery and Trauma Centre Copenhagen University Hospital—Rigshospitalet Copenhagen Denmark

## Abstract

**Background:**

Thrombocytopenia is frequent in adult intensive care unit (ICU) patients and may contribute to adverse outcomes, including bleeding, morbidity and mortality. To reduce the risk of bleeding, platelet transfusions are commonly used, yet transfusion thresholds vary, and the evidence base is very limited. Randomised clinical trials investigating platelet transfusion strategies in ICU patients are therefore a high research priority. With this survey, we aim to assess ICU doctors' willingness to randomise adult ICU patients to restrictive versus liberal platelet transfusion thresholds across clinically relevant scenarios in a future randomised trial.

**Methods:**

We will conduct an international, web‐based survey targeting ICU doctors within an established research network. The survey includes questions on respondent characteristics and willingness to enrol patients with an indication for platelet transfusion across a range of transfusion thresholds in different clinical scenarios, including non‐bleeding patients, those undergoing invasive procedures, and those with bleeding. The survey has been pilot‐tested and refined twice before distribution. We will summarise results using descriptive statistics and conduct stratified analyses by country and ICU type.

**Conclusions:**

This international survey will assess ICU doctors' willingness to randomise adult ICU patients to restrictive versus liberal platelet transfusion strategies across multiple clinical scenarios.

## 1 Introduction

Thrombocytopenia is commonly defined as a platelet count below 150 × 10^9^/L and is frequently observed in intensive care unit (ICU) patients [1, 2]. Its aetiology is often multifactorial, encompassing conditions and interventions that impair platelet production, increase platelet consumption or cause destruction or sequestration, as well as haemodilution [3]. Observational studies have linked thrombocytopenia to worse short‐ and long‐term outcomes in ICU patients, including increased bleeding, morbidity and mortality [4, 5, 6].

Platelet transfusions are commonly administered in the ICU, both prophylactically and before invasive procedures, and to treat bleeding [7]. Hence, approximately 10% of all acutely admitted adult ICU patients receive platelet transfusions [4]. Despite their frequent use, transfusion practices vary considerably between centres, and the evidence base supporting current practice remains uncertain [8, 9, 10]. Furthermore, platelet products are limited in availability. Thus, robust randomised clinical trials evaluating platelet transfusion thresholds in ICU patients have been recognised as a research priority by the *European Society of Intensive Care Medicine* and are urgently needed to optimise platelet use in the ICU [9, 10].

The outlined survey aims to assess whether intensive care physicians working in relevant ICUs would be willing to randomise adult ICU patients to restrictive versus liberal platelet transfusion thresholds across different clinical scenarios in a future clinical trial.

## 2 Methods

### 2.1 Study Design, Data Collection, Approvals, and Reporting

The survey is designed based on the *Consensus‐Based Checklist for Reporting of Survey Studies* (*CROSS*) and has undergone two rounds of pilot testing. The first round involved six ICU physicians and researchers, followed by a second round with five other researchers, all at the coordinating centre, Department of Intensive Care, Copenhagen University Hospital—Rigshospitalet, Denmark. The survey has been revised accordingly before distribution.

Participation will be voluntary, and no financial incentives will be offered. Activation of the survey link and submission of a completed questionnaire constitute informed consent. Respondents' email addresses will be obtained, if possible, upon request from the designated local investigators, to verify the uniqueness of responses before calculating the response rate.

Once data collection is complete, email addresses will be removed from the dataset to ensure participant confidentiality. In cases of missing data, respondents will be contacted for further clarification or additional information.

Data will be collected and managed using *Research Electronic Data Capture* (*REDCap*) [11, 12], hosted by the Capital Region of Denmark. *REDCap* is a secure, web‐based software platform designed to support data capture for research studies. It includes built‐in data validation and audit trails to safeguard data integrity and participant confidentiality, with encryption and secure storage.

We have obtained approval from the Legal Department of Scientific Research in the Capital Region of Denmark (approval number: p‐2025‐20,473). Ethical and additional regulatory approvals are not applicable, as no patient‐level data will be collected. The only potentially identifiable information obtained from participating ICU physicians will be their work email addresses. These will be used solely for response validation and will not be stored beyond the validation process, nor will they be shared or distributed.

Study results will be reported in accordance with the *CROSS* checklist. A completed checklist for this protocol is provided in Data S1 [13]. The study protocol has been publicly registered to ensure transparency [14]. At the time of submission of this protocol manuscript, the survey has not yet been distributed.

### 2.2 Survey Description

The survey comprises two parts. The first part consists of questions that collect information on respondents' characteristics, including their professional background, clinical setting and work email addresses. The second part includes questions that assess respondents' willingness to participate in a clinical trial evaluating a restrictive versus a liberal platelet transfusion strategy across different clinical scenarios and low versus high platelet transfusion thresholds. The clinical scenarios include:

- Non‐bleeding adult ICU patients with thrombocytopenia and no planned invasive procedures.
- Non‐bleeding adult ICU patients with thrombocytopenia who are scheduled for ultrasound‐guided central venous catheter placement, bronchoscopy with or without lavage, ultrasound‐guided drainage of pleural or abdominal fluid collections, dilatational tracheostomy, spinal puncture, epidural catheter placement, minor surgery, major surgery and major vascular and thoracic surgery.
- ICU patients with thrombocytopenia and minor bleeding.
- ICU patient with thrombocytopenia and major controlled or non‐massive bleeding.

The full survey is provided in Data S2.

### 2.3 Survey Distribution

The survey will be distributed through the established international network for intensive care research, focusing on sites currently participating in, or planning to participate in, the *Intensive Care Platform Trial* (*INCEPT*) [15]. Intensive care physicians working in the relevant ICUs will be invited to participate, as they constitute the target population expected to enrol and treat participants in a forthcoming *INCEPT* domain (sub‐trial). Responses to the survey will therefore directly inform the design and feasibility of the upcoming platform trial domain.

Each participating department will appoint a designated local site investigator. The local investigator will be responsible for providing the coordinating centre with the work email addresses of eligible ICU doctors who will participate in the survey. The coordinating site will subsequently distribute an email containing a unique link to the online survey to each invited ICU physician. Alternatively, if preferred, local investigators may request a general link to the survey and coordinate distribution to ICU doctors at their site themselves while tracking the number of invited survey participants. To increase the response rate, non‐responders will receive up to three email reminders before database closure.

### 2.4 Statistics

We will present descriptive data, reporting counts and percentages for the categorical variables, and conduct stratified analyses by country and by type of ICU (surgical/medical/mixed/neurological/cardio‐thoracic/other). We will report the response rate and proportion of missing data, and analyses will be conducted on complete cases only. No formal sample size calculation has been performed. However, based on a similar previous survey, we anticipate approximately 400 respondents [16], which we consider sufficient to provide robust and informative insights.

## 3 Discussion

The outlined survey will provide insights into ICU doctors' willingness to randomise adult ICU patients in a clinical trial comparing a restrictive versus a liberal platelet transfusion strategy. It will directly inform the design of the upcoming *INCEPT‐Platelets* domain (i.e., sub‐trial) within the investigator‐initiated, international, pragmatic, randomised, embedded, multifactorial, adaptive platform trial *INCEPT* [15].

### 3.1 Strengths and Limitations

A major strength of this survey is its direct alignment with the clinicians expected to participate in the upcoming *INCEPT‐Platelets* domain. By targeting ICU doctors likely to enrol and treat participants in the upcoming trial, the survey will provide valuable insights into the design, acceptability, and feasibility of the domain. The survey focuses on specific clinical scenarios and platelet count thresholds, thereby enhancing its relevance to real‐world practice and minimising ambiguity in interpreting the results.

There are several limitations to consider. First, response bias may occur, as respondents may give answers they believe are more favourable or expected. Second, participation will be voluntary rather than random, which may lead to self‐selection or non‐response bias. Third, since most respondents are likely to be from Denmark, the survey results could be skewed; however, stratified analysis by country will be used if necessary. Fourth, limiting analyses to complete cases may be problematic if incomplete responses are present. Nevertheless, because all survey questions are mandatory and must be answered before submission, missing data should not affect the analyses. Still, a low to moderate response rate could introduce bias. Finally, limiting distribution to the INCEPT network may reduce the external validity of the findings.

## 4 Conclusion

The outlined survey will assess ICU doctors' willingness to randomise adult ICU patients to restrictive versus liberal platelet transfusion strategies across multiple clinical scenarios, thereby informing the design of the upcoming *INCEPT‐Platelets* domain.

## Author Contributions

Conceptualisation: all authors. Methodology: all authors. Project administration: J.A.B. Supervision: C.T.A., A.G., M.H.M., J.S. and A.P. Writing – original draft: J.A.B. Writing – review and editing: all authors.

## Funding

The authors have nothing to report.

## Conflicts of Interest

All authors are involved in designing the future *INCEPT‐Platelets* domain within the *Intensive Care Platform Trial* (*INCEPT*; www.incept.dk), which investigates platelet transfusion strategies. *INCEPT* is funded by the Novo Nordisk Foundation and Sygeforsikringen ‘danmark’, with additional support from Grosserer Jakob Ehrenreich og Hustru Grete Ehrenreichs Fond, Dagmar Marshalls Fond and Savværksejer Jeppe Juhl og hustru Ovita Juhls Mindelegat.

## Supporting information


**Data S1:** Completed Checklist for Reporting Of Survey Studies (CROSS).

**Data S2:** The full survey as it will be implemented and presented to participants in REDCap.

## Data Availability

Data sharing not applicable to this article as no datasets were generated or analysed during the current study.
