# Supplementary material for: ICU Clinicians' View on Platelet Transfusion Thresholds for a Future Trial—Protocol for an International Survey
Source: Acta Anaesthesiol Scand. 2026 May 19;70:e70263. doi: 10.1111/aas.70263 (PMC13184580; doi:10.1111/aas.70263)
Supplement: Supplementary file 2 — Data S2: The full survey as it will be implemented and presented to participants in REDCap. [file AAS-70-0-s002.pdf]

ICU clinicians' view on platelet transfusion thresholds for a future trial – protocol for an international survey

Jehad Ahmad Barakji[1], Carl Thomas Anthon[1], Anders Granholm[1,2], Morten Hylander Møller[1,3], Jakob Stensballe[3,4], Anders Perner[1,3]

[1] Department of Intensive Care, Copenhagen University Hospital - Rigshospitalet, Copenhagen, Denmark

[2] Section of Biostatistics, Department of Public Health, University of Copenhagen, Copenhagen, Denmark

[3] Department of Clinical Medicine, University of Copenhagen, Copenhagen, Denmark

[4] Department of Anaesthesiology, Surgery and Trauma Centre, Copenhagen University Hospital - Rigshospitalet, Copenhagen, Denmark

Supplement S2

This document contains the full survey as it will be implemented in REDCap and presented to participants. The survey is scenario-based and designed to reflect common ICU clinical situations, with a focus on platelet transfusion decision-making.

INCEPT-Platelets survey

E-mail

Please enter your work email address.

(* must provide value)

Demography

1) What is your clinical position?

(* must provide value)

- Medical doctor (specialist)
- Medical doctor (non-specialist)

Definitions:

Medical doctor (specialist): a doctor certified in a medical or surgical specialty (e.g., anaesthesiology, cardiology or surgery).

Medical doctor (non-specialist): a doctor without specialty certification, providing specialised care under supervision.

2) In which country do you currently work?
(* must provide value)

- Austria
- Denmark
- Estonia
- Finland
- Iceland
- Latvia
- Norway
- Sweden
- Switzerland
- The Netherlands

3) Please select your type of hospital
(* must provide value)

- Public general hospital
- Public specialist hospital
- Private general hospital
- Private specialist hospital

Definitions:

General hospital: hospital or medical centre that can provide licensed physicians in internal medicine, paediatrics, obstetrics, gynaecology, orthopaedics, general surgery, and other supporting medical services.

Specialist hospital: hospital that can provide similar services as described for general hospitals, and in addition does specialised care such as neurosurgery, cardiac surgery, or transplantation.

4) Please specify your type of intensive care unit (ICU)
(* must provide value)

- Surgical ICU
- Medical ICU
- Mixed ICU admitting both surgical and medical patients
- Neurological/neurosurgical ICU
- Cardio-thoracic ICU
- Other (please specify)

Definitions:

Surgical ICU: unit specialised in treating patients that are originating from the operating or recovery room or if surgical complications lead to ICU admission. Including trauma patients.

Medical ICU: unit with a broad patient profile, not specialised in treating a specific patient group or disease.

Mixed ICU: unit containing surgical ICU patients and medical ICU patients.

Neurological/neurosurgical ICU: unit specialised in treating patients with traumatic brain injury, stroke, intracranial haemorrhage, brain tumour, spinal cord injury, and providing post-operative care following neurosurgical procedures.

Cardio-thoracic ICU: unit specialised in treating patients with heart failure, severe arrhythmia, acute coronary syndrome and providing post-operative care following cardiac and thoracic surgery.

4a) Please specify type of intensive care unit (ICU)

Text field

Clinical scenarios

You will now be introduced to three main clinical scenarios with one or more questions. For each scenario, please consider that you are about to potentially enrol a patient in your ICU in a trial comparing lower vs. higher platelet count thresholds for platelet

transfusion. For each question, indicate which platelet threshold pairs for transfusion you would be willing to randomise patients between in such a trial.

The scenarios are:

1) Non-bleeding adult ICU patient with thrombocytopenia, not receiving antiplatelet medication and no planned invasive procedure (Q5).

2) Non-bleeding adult ICU patient with thrombocytopenia and not receiving antiplatelet medication undergoing an invasive procedure or surgery (Q5-Q14).

3) Bleeding adult ICU patient with thrombocytopenia and not receiving antiplatelet medication (Q15-Q16).

Scenario 1: Non-bleeding adult ICU patient with thrombocytopenia, not receiving antiplatelet medication and no planned invasive procedure or surgery

5) **In a non-bleeding adult ICU patient with no planned invasive procedure (10^9 cells/L)?**

	Restrictive		Liberal	
a.	No platelet transfusion	vs.	<20	(y/n)
b.	<10	vs.	<30	(y/n)
c.	<20	vs.	<40	(y/n)
d.	<30	vs.	<60	(y/n)
e.	<40	vs.	<80	(y/n)
f.	<50	vs.	<100	(y/n)

Scenario 2: Non-bleeding adult ICU patient with thrombocytopenia and not receiving antiplatelet medication undergoing an invasive procedure or surgery

6) **Undergoing ultrasound-guided central venous catheter placement in the jugular vein or the femoral vein (including vascular access for renal replacement therapy) in the ICU (10^9 cells/L)?**

	Restrictive		Liberal	
a.	No platelet transfusion	vs.	<20	(y/n)
b.	<10	vs.	<30	(y/n)
c.	<20	vs.	<40	(y/n)
d.	<30	vs.	<60	(y/n)
e.	<40	vs.	<80	(y/n)
f.	<50	vs.	<100	(y/n)

7) **Undergoing bronchoscopy with or without lavage in the ICU (10^9 cells/L)?**

	Restrictive		Liberal	
a.	No platelet transfusion	vs.	<20	(y/n)
b.	<10	vs.	<30	(y/n)
c.	<20	vs.	<40	(y/n)
d.	<30	vs.	<60	(y/n)
e.	<40	vs.	<80	(y/n)
f.	<50	vs.	<100	(y/n)

8) **Undergoing ultrasound-guided drainage of pleural- or abdominal fluid collections including ascites (10^9 cells/L)?**

	Restrictive		Liberal	
a.	No platelet transfusion	vs.	<20	(y/n)
b.	<10	vs.	<30	(y/n)
c.	<20	vs.	<40	(y/n)
d.	<30	vs.	<60	(y/n)
e.	<40	vs.	<80	(y/n)
f.	<50	vs.	<100	(y/n)

9) **Undergoing dilatational tracheostomy in the ICU (10^9 cells/L)?**

	Restrictive		Liberal	
a.	No platelet transfusion	vs.	<20	(y/n)
b.	<10	vs.	<30	(y/n)
c.	<20	vs.	<40	(y/n)
d.	<30	vs.	<60	(y/n)
e.	<40	vs.	<80	(y/n)
f.	<50	vs.	<100	(y/n)

10) **Undergoing spinal puncture in the ICU (e.g., for suspected central nervous system infection) (10^9 cells/L)?**

	Restrictive		Liberal	
a.	No platelet transfusion	vs.	<20	(y/n)
b.	<10	vs.	<30	(y/n)

c.	<20	vs.	<40	(y/n)
d.	<30	vs.	<60	(y/n)
e.	<40	vs.	<80	(y/n)
f.	<50	vs.	<100	(y/n)

11) **Undergoing epidural catheter placement in the ICU (10^9 cells/L)?**

	Restrictive		Liberal	
a.	No platelet transfusion	vs.	<20	(y/n)
b.	<10	vs.	<30	(y/n)
c.	<20	vs.	<40	(y/n)
d.	<30	vs.	<60	(y/n)
e.	<40	vs.	<80	(y/n)
f.	<50	vs.	<100	(y/n)

12) **Undergoing minor surgery (e.g., wound dressing changes, wound closure, re-look laparotomy, or soft tissue surgery, but excluding brain and spinal cord surgery) (10^9 cells/L)?**

	Restrictive		Liberal	
a.	No platelet transfusion	vs.	<20	(y/n)
b.	<10	vs.	<30	(y/n)
c.	<20	vs.	<40	(y/n)
d.	<30	vs.	<60	(y/n)
e.	<40	vs.	<80	(y/n)
f.	<50	vs.	<100	(y/n)

13) **Undergoing major surgery (e.g., emergency laparotomy, major fracture surgery, or major soft tissue surgery, but excluding brain and spinal cord surgery) (10^9 cells/L)?**

	Restrictive		Liberal	
a.	No platelet transfusion	vs.	<20	(y/n)
b.	<10	vs.	<30	(y/n)
c.	<20	vs.	<40	(y/n)
d.	<30	vs.	<60	(y/n)
e.	<40	vs.	<80	(y/n)

f.	<50	vs.	<100	(y/n)

14) **Undergoing major vascular and thoracic surgery (e.g., open abdominal aortic aneurysm repair, endovascular aneurysm repair, valve replacement or repair, or video-assisted thoracoscopic surgery for major lung resection) (10^9 cells/L)?**

	Restrictive		Liberal	
a.	No platelet transfusion	vs.	<20	(y/n)
b.	<10	vs.	<30	(y/n)
c.	<20	vs.	<40	(y/n)
d.	<30	vs.	<60	(y/n)
e.	<40	vs.	<80	(y/n)
f.	<50	vs.	<100	(y/n)

Scenario 3: Bleeding adult ICU patient with thrombocytopenia and not receiving antiplatelet medication

15) **Adult ICU patient with minor bleeding (e.g., minor haematuria, minor melena, minor puncture site bleeding, minor epistaxis, or minor oropharyngeal bleeding) (10^9 cells/L)?**

	Restrictive		Liberal	
a.	No platelet transfusion	vs.	<20	(y/n)
b.	<10	vs.	<30	(y/n)
c.	<20	vs.	<40	(y/n)
d.	<30	vs.	<60	(y/n)
e.	<40	vs.	<80	(y/n)
f.	<50	vs.	<100	(y/n)

16) **Adult ICU patient with major controlled/non-massive bleeding requiring red blood cell transfusion and/or surgical/radiological/procedural interventions (e.g., gastrointestinal-, pulmonary-, soft tissue-, joint-, muscle-, or urogenital bleeding, but excluding intracerebral and spinal bleeding) (10^9 cells/L)?**

	Restrictive		Liberal	
a.	No platelet transfusion	vs.	<20	(y/n)
b.	<10	vs.	<30	(y/n)
c.	<20	vs.	<40	(y/n)
d.	<30	vs.	<60	(y/n)
e.	<40	vs.	<80	(y/n)
f.	<50	vs.	<100	(y/n)
